# Prenatal Exposure to Mixtures of Phthalates, Parabens, and Other Phenols and Obesity in Five-Year-Olds in the CHAMACOS Cohort

**DOI:** 10.3390/ijerph18041796

**Published:** 2021-02-12

**Authors:** Kimberly Berger, Carly Hyland, Jennifer L. Ames, Ana M. Mora, Karen Huen, Brenda Eskenazi, Nina Holland, Kim G. Harley

**Affiliations:** 1 Sequoia Foundation, La Jolla, CA 92037, USA; kimberly.berger@cdph.ca.gov; 2 Center for Environmental Research and Children’s Health (CERCH), School of Public Health, University of California at Berkeley, Berkeley, CA 94704, USA; chyland@berkeley.edu (C.H.); animora@berkeley.edu (A.M.M.); khuen@berkeley.edu (K.H.); eskenazi@berkeley.edu (B.E.); ninah@berkeley.edu (N.H.); 3 Division of Research, Kaiser Permanente Northern California, Oakland, CA 94612, USA; jennifer.l.ames@kp.org; 4 Central American Institute for Studies on Toxic Substances (IRET), Universidad Nacional, Heredia 40101, Costa Rica

**Keywords:** obesity, phthalates, parabens, phenols, endocrine disruptors, children’s health, mixtures, Bayesian

## Abstract

Exposures to phthalates, parabens, and other phenols are often correlated due to their ubiquitous use in personal care products and plastics. Examining these compounds as a complex mixture may clarify inconsistent relationships between individual chemicals and childhood adiposity. Using data from the Center for the Health Assessment of Mothers and Children of Salinas (CHAMACOS) study, a longitudinal cohort of children in Salinas Valley, California (*n* = 309), we examined biomarkers of 11 phthalate metabolites and 9 phenols, including several parabens and bisphenol A, measured in maternal urine at two time points during pregnancy. We measured child height and weight at age five to calculate the body mass index (BMI) *z*-scores and overweight/obesity status. The association between prenatal urinary concentrations of biomarkers with the childhood BMI *z*-score and overweight/obesity status was analyzed using single-pollutant models and two mixture methods: Bayesian hierarchical modeling (BMH) and Bayesian kernel machine regression (BKMR). Urinary concentrations of monoethyl phthalate, monocarboxy-isononly phthalate (metabolites of diethyl phthalate and di-isodecyl phthalate, respectively), and propylparaben were consistently associated with an increased BMI *z*-score and overweight/obesity status across all modeling approaches. Higher prenatal exposures to the cumulative biomarker mixture also trended with greater childhood adiposity. These results, robust across two methods that control for co-pollutant confounding, suggest that prenatal exposure to certain phthalates and parabens may increase the risk for obesity in early childhood.

## 1. Introduction

An estimated 18.5% of all US children were classified as obese in 2016, up from 15.6% in 2006, with even higher rates among Hispanic children [1,2]. Obesity in early childhood is a risk factor for obesity in adulthood, as well as for serious health conditions such as diabetes mellitus, heart disease, and depression [3]. It has been hypothesized that exposure to endocrine-disrupting chemicals (EDCs) may play a role in the obesity epidemic [4], and exposure during the prenatal period may be a critical window for childhood and adult obesity, and associated health effects [5].

Phthalates, parabens, and phenols are EDCs [4,6] that are commonly used in plastics, personal care products, and other consumer products. Though these chemicals are readily metabolized and are typically non-persistent in the body, exposures are often chronic and ubiquitous. Data from the 2013–2016 National Health and Nutrition Examination Survey (NHANES) indicates that urinary biomarkers of most of these chemicals are detected in over 90% of the US population, with higher concentrations observed in women [7]. Toxicological and epidemiological evidence has raised concern about these chemicals’ endocrine-disrupting and possibly obesogenic effects [8]. Many phthalates, parabens, and phenols appear to cross the placenta [9] and may disrupt pathways of endocrine signaling and inflammatory responses [10], possibly through changes to fetal epigenetic mechanisms [11,12] and/or action on peroxisome proliferator-activated receptors [13].

Low-molecular-weight (LMW) phthalates, which are found in cosmetics, lotions, scents, and nail polish [14], and high-molecular-weight (HMW) phthalates, which are found in flexible plastics [14], have been largely associated with several measures of childhood obesity [15]. In utero exposure to some parabens, compounds found in cosmetics, paper products, and pharmaceuticals [16], has been associated with similar childhood outcomes [17,18], as has 2,5-dichlorophenol [19,20], a metabolite of 1,4-dichlorobenzene, which is used in deodorizers and moth balls [21]. Studies, however, have not been entirely consistent [19,22], and those conducted later in childhood, often with cross-sectional designs, have offered more mixed findings [23,24,25,26]. Evidence is also less conclusive for phenols such as bisphenol A (BPA) [4], found in hard plastics and food packaging [27]; triclosan [17,20,28], an antimicrobial found in toothpaste, deodorant, and some textiles [29]; benzophenone-3 [19,20], a sunscreen [30]; and 2,4-dichlorophenol [22,31], a product of pesticide and chlorinated compound manufacturing that is also a photo-degradant of triclosan [32].

Because these chemicals are found in common consumer products, and because products often contain multiple chemicals, individuals are regularly exposed to mixtures of these chemicals. Methods that can examine these exposures as complex mixtures can help address co-pollutant confounding and potential interactions among chemicals. In a previous study, we used single-pollutant models to explore the relationship between phthalates and measures of childhood adiposity and found several positive associations in children aged 5 to 12 years old that were robust in mixture analysis using Bayesian kernel machine regression (BKMR) when applied to the data of the 12-year-olds [33]. Three prior studies in other populations have conducted mixture modeling of phthalates, parabens, and other phenols. Two studies in NHANES found BPA and several phthalate biomarkers to be positively associated with measures of adiposity in both children and adults, with 2,4-dichlorophenol also associated in children [34,35] after accounting for multiple exposures. Only one previous study has examined mixtures of these biomarkers during pregnancy, a period of high susceptibility. In that study, prenatal biomarkers of several phthalates in a Spanish cohort were unexpectedly associated with a lower risk for being overweight/obese at age seven years, while BPA showed no association [36].

The current study aims to examine the relationship of prenatal exposure to three chemical classes (phthalates, parabens, and phenols), as measured by maternal prenatal urinary biomarkers, in relation to adiposity of children at age five. This younger age avoids the potential mediating effects of puberty onset that might be present at later ages (e.g., seven or nine years). A previous analysis of this cohort examined the body mass index (BMI) trajectory over ages 2 to 14 and found that the BMI *z*-score remained steady after age 5, with the BMI gradually increasing in the population overall [37]. We will employ two mixture methods, Bayesian hierarchical modeling (BHM) and BKMR, that allow us to explore complex relationships among multiple biomarkers of phthalates, parabens, and other phenols and account for co-pollutant confounding.

## 2. Materials and Methods

### 2.1. Study Population

The Center for the Health Assessment of Mothers and Children of Salinas (CHAMACOS) is a longitudinal cohort study that began in 1999–2000 with the enrollment of 601 pregnant women living in Salinas Valley, California, an agricultural area with a large population of Mexican immigrants. Women were eligible for enrollment if they were over 18, planning to give birth at the county hospital, spoke English or Spanish, and qualified for Medicaid (low-income health insurance). Women were recruited during prenatal care visits at one of several partner clinics in the area. The study continues to follow up participants and their children, and data have been collected every few years since enrollment. We limited the current analysis to participants with complete data on prenatal biomarkers of exposure to phthalates, parabens, and other phenols; height and weight measurements at the five-year assessment; and relevant covariates (listed below), because BHM and BKMR can only be conducted with nonmissing data. Of the 601 participants enrolled, 537 stayed in the study through the birth of a live-born child and 331 children participated in the follow-up visit at age five. Of these, 314 had complete data on all biomarkers and 312 had complete covariate data. From those, a randomly selected twin from each twin pair was dropped (*n* = 3 dropped twins) in order to maintain independence in outcome measurements, resulting in a final population of 309. Participants included in the current study were overall similar in demographics to participants who were lost to follow-up, though the included children were less likely to have been low birth weight and their mothers tended to be older, had lived in the U.S. longer, and were more likely to be overweight or obese than those lost to follow-up (Supplemental Table S1).

The Institutional Review Board (IRB) of the University of California, Berkeley approved all study activities, and informed consent was obtained from mothers at all study visits. The Centers for Disease Control and Prevention (CDC) IRB deferred approval to the IRB at the University of California, Berkeley (IRB approval number: 2010-03-949).

Maternal demographic and covariate data were obtained from mothers via structured interviews in English or Spanish at the two pregnancy visits (mean ± SD: 14.0 ± 4.8 and 26.9 ± 2.4 weeks’ gestation), and childhood fast-food intake information was obtained from the maternal interview during the visit at age five. Infant birth weight was abstracted from medical records. Measured height and self-reported pre-pregnancy weight were used to calculate the pre-pregnancy BMI. If mothers did not report the pre-pregnancy weight (*n* = 38), it was imputed from the weight at the first prenatal care visit.

### 2.2. Biomarker Measurements

At both pregnancy visits, urine samples were collected from mothers in polypropylene urine cups, then aliquoted into glass vials, and stored at −80 °C until shipment for analysis at the CDC in Atlanta, Georgia.

Urinary biomarkers were measured using solid-phase extraction coupled with isotope dilution high-performance liquid chromatography–electrospray ionization–tandem mass spectrometry. Analytic methods for phthalates [38] and phenols [39] have been published previously. We quantified concentrations of three LMW phthalate metabolites: monoethyl phthalate (MEP), a metabolite of diethyl phthalate (DEP); mono-n-butyl phthalate (MBP), a metabolite of di-n-butyl phthalate (DBP); and mono-isobutyl phthalate (MiBP), a metabolite of diisobutyl phthalate (DiBP). We also quantified concentrations of eight HMW phthalates: monobenzyl phthalate (MBzP), a metabolite of benzyl butyl phthalate (BBzP); four metabolites of di(2-ethylhexyl) phthalate (DEHP) (mono-2-ethylhexyl phthalate (MEHP), mono-(2-ethyl-5-hydroxyhexyl) phthalate (MEHHP), mono-(2-ethyl-5-oxohexyl) phthalate (MEOHP), and mono-(2-ethyl-5-carboxypentyl) phthalate (MECPP)); monocarboxyoctyl phthalate (MCOP), a metabolite of di-isononyl phthalate (DiNP); monocarboxy-isononly phthalate (MCNP), a metabolite of di-isodecyl phthalate (DiDP); and mono(3-carboxypropyl) phthalate (MCPP), a metabolite of several HMW phthalates and a minor metabolite of DBP. We quantified three parabens: methylparaben, butylparaben, propylparaben; however, butylparaben was not included in statistical analyses due to the low detection frequency (55% and 58% were below the limit of detection (LOD) in the first and second samples, respectively). We measured five phenols: triclosan, 2,4-dichlorophenol, 2,5-dichlorophenol, benzophenone-3, and BPA. Concentrations were reported in ng/mL of urine. LODs ranged from 0.2 ng/mL to 2.3 ng/mL. Concentrations below the LOD were assigned instrumental reading values, if available, or an imputed value below the LOD selected randomly from the log-normal distribution using the maximum likelihood estimation [40]. 

The urinary specific gravity was measured using a hand-held refractometer (National Instrument Company Inc., Baltimore, MD, USA). We corrected for urinary dilution using the following formula: (analyte concentration × 0.24)/(sample specific gravity − 1), where 0.24 is the median value for specific gravity in our population [41]. We imputed the urinary specific gravity based on urinary creatinine concentrations for 58 women missing specific gravity measurements by conducting a univariate regression of nonmissing logged creatinine on nonmissing logged specific gravity and using the resulting regression equation to estimate missing specific gravity values using creatinine concentrations.

### 2.3. Anthropometric Measurements

At age five, each child’s standing height without shoes was measured in triplicate using a stadiometer (Seca 222, Chino, CA, USA) and then averaged. Weight was measured without shoes or jackets using an electronic scale (Tanita 1582, Arlington Heights, IL, USA).

### 2.4. Statistical Analysis

The two pregnancy measurements for each biomarker were averaged and log2-transformed. If a woman had only one measurement (*n* = 13 for phthalates, *n* = 20 for parabens/other phenols), it was used in place of a pregnancy average. The molar concentrations of the four metabolites of DEHP (MEHP, MEHHP, MEOHP, MECPP) were summed into a ΣDEHP variable because they are highly correlated and together account for at least 60% of DEHP exposure [42].

The BMI was calculated as weight/height^2^ (kg/m^2^) and categorized according to the CDC’s age- and sex-specific percentiles into underweight, normal weight, overweight, and obese [43]. The BMI *z*-score was calculated using CDC norms by age and sex [44]. We created an indicator variable for children who were overweight or obese versus those who were normal weight or underweight.

While not the main focus of this paper, we conducted single-biomarker linear and logistic models for the childhood BMI *z*-score and overweight/obesity status, respectively, for comparison to results from the mixture methods; the current sample is slightly different from that of our previous paper [33] as we limited the sample to participants with complete data on all biomarkers since BHM and BKMR both necessitate no missing data. Separate models were conducted for each biomarker.

We used a Bayesian modeling framework to examine exposure–outcome associations in order to address co-pollutant confounding and complex relationships among biomarkers. First, we implemented two-stage BHM to calculate β effect estimates and odds ratios (ORs) in models that included all 15 prenatal biomarkers simultaneously. In the first stage, we regressed the continuous BMI *z*-score and binary normal vs. overweight/obesity outcomes on the 15 prenatal biomarker concentrations and covariates in linear and logistic regression models, respectively. In the second stage, we modeled the first-stage effect estimates as a function of their exchangeability matrix, *Z*, coefficient vector, *π*, and residual error, *δ*, which was assumed to be normally distributed with mean 0 and variance *τ*^2^, as *β* = *Zπ* + *δ*. We constructed a *Z* matrix that included indicator variables (0/1), assigning each biomarker to one of four chemical classes (LMW phthalates, HMW phthalates, parabens, or other phenols), incorporating our a priori expectation that biomarkers from the same class would exert similar effects on the outcome. For the linear BMI *z*-score outcome, we selected a value of *τ* that reflected our prior belief that the *β* estimate would lie within ±0.5 SD of the mean in our population; however, we conducted sensitivity analyses with values of *τ* reflecting the possibility of the *β* estimate lying within ±0.25 SD or ±1.0 SD of the mean. Following previous applications of BHM for binary outcomes [45,46], we set *τ* = 0.35 for the binary normal vs. overweight/obesity outcome, reflecting our prior belief that ORs would lie within a fourfold range (e.g., from 0.5 to 2.0).

We also used component-wise BKMR to model the childhood BMI *z*-score and overweight/obesity status as flexible, non-parametric, non-additive kernel functions of all urinary biomarker concentrations. The program outputs several types of figures: we present the univariate figures, which represent the higher-dimensional modeling in a two-dimensional graph of each scaled biomarker’s association with the outcome, holding all other biomarkers at their medians, and figures for the overall effect of the mixture, representing the predicted probability of the outcome at quantiles of the total concentration of all scaled biomarkers.

In all models, we controlled for maternal age at delivery, maternal education, years lived in the U.S. at delivery, poverty status during pregnancy (at or below vs. above poverty threshold), and the childhood frequency of fast-food intake at age five (<1 time per week, 1–2 times per week, and 3+ times per week). These covariates were chosen a priori via the directed acyclic graph. We controlled for consumption of fast food at age five as a strong predictor of the outcome. Fast food is a significant exposure source for HMW phthalates and BPA, and consumption may be associated with obesity [47,48]. This covariate may also function as a proxy for maternal consumption during pregnancy because data on fast-food intake during pregnancy were not collected. We conducted sensitivity analyses excluding consumption of fast food at age five and also conducted unadjusted analyses.

Data cleaning was performed using Stata 14 (College Station, TX, USA). All analyses were performed in R (Vienna, Austria).

## 3. Results

Almost all mothers were Latina (98%), tended to be of low-income and low-education levels, and were mostly under the age of 30 (mean age = 26.7 years, SD = 5.3 years) (Table 1). Mothers tended to have lived in the U.S. for five or fewer years prior to delivery (46%) and were mostly overweight or obese (66%), and most households were living at or below 100% of the federal poverty threshold (63%). Very few children (~4%) were born with a low birth weight. At age five, most children consumed fast food one to two times a week (61%), and 46% were normal weight, 21% were overweight, and 33% were obese.

**Figure 1:**
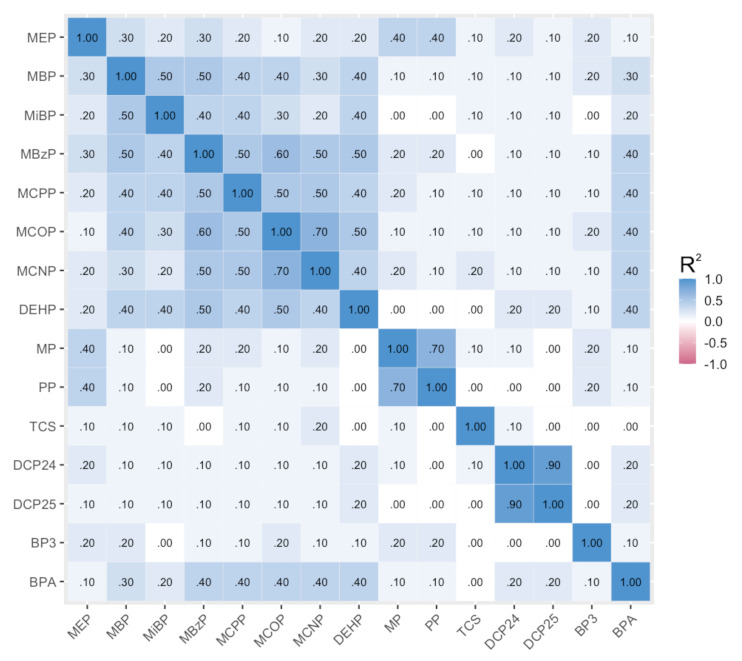
Correlations of log2 specific-gravity-corrected phthalate, paraben, and other phenol urinary concentrations of CHAMACOS mothers during pregnancy.

All biomarkers were detected in at least 80% of samples, with the exception of triclosan, which was detected in 71% of samples at the first visit and 75% at the second visit (Table 2). Figure 1 shows the correlation of exposure biomarkers, averaged across the two prenatal measurements. Urinary concentrations of 2,4-dichlorophenol and 2,5-dichlorophenol were the most highly correlated (*R*^2^ = 0.90, *p* < 0.001), followed by methylparaben and propylparaben (*R*^2^ = 0.70, *p* < 0.001), which were also moderately correlated with MEP (methylparaben *R*^2^ = 0.40, *p* = 0.06; propylparaben *R*^2^ = 0.40, *p* = 0.05). Most phthalates were moderately correlated with each other, as were BPA and the HMW phthalates.

Table 3 shows results from both single-biomarker linear and logistic regressions and Bayesian hierarchical models. In single-biomarker models, a twofold increase in prenatal concentrations of MEP (β: 0.10, 95% confidence interval (95% CI): 0.04, 0.17), methylparaben (β: 0.08, 95% CI: 0.01–0.16), and propylparaben (β: 0.06, 95% CI: 0.02, 0.10) was associated with a higher BMI *z*-score at age five. Prenatal concentrations of MBP (β: 0.07, 95% CI: −0.04, 0.17) and MCNP (β: 0.10, 95% CI: −0.02, 0.23) were also associated with a higher BMI *z*-score; however, confidence intervals contained null. In Bayesian hierarchical models, we observed associations of prenatal concentrations of MEP (β: 0.08, 95% credible interval (95% CrI): 0.00, 0.16), MCNP (β: 0.15, 95% CrI: −0.02, 0.32), and propylparaben (β: 0.04, 95% CrI: −0.02, 0.10) and a higher childhood BMI *z*-score.

For overweight/obesity status, a twofold increase in MEP (odds ratio (OR): 1.23, 95% CI: 1.08, 1.42), methylparaben (OR: 1.22, 95% CI: 1.06, 1.40), and propylparaben (OR: 1.14, 95% CI: 1.05, 1.24) was again associated in single-biomarker models. Higher prenatal concentrations of 2,4-dichlorophenol (OR: 1.07, 95% CI: 0.95, 1.22) and 2,5-dichlorophenol (OR: 1.05, 95% CI: 0.96, 1.14) were also associated with slightly higher odds of being overweight/obese in single-pollutant models; however, confidence intervals contained null. In Bayesian hierarchical models, we observed higher odds of overweight/obesity status in association with a twofold increase in prenatal MEP (OR: 1.2, 95% CrI: 1.02, 1.42), MCNP (OR: 1.37, 95% CrI: 1.01, 1.89), and propylparaben (OR: 1.08, 95% CrI: 0.97, 1.22) concentrations.

Table 4 shows the posterior inclusion probabilities (PIP) from the BKMR analysis for both outcomes. For both outcomes, propylparaben is most influential on the outcome, followed by MEP and methylparaben.

**Figure 2:**
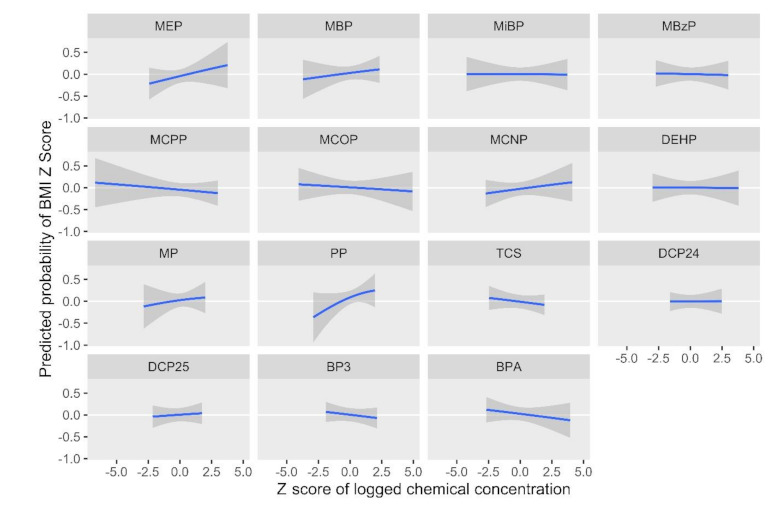
Predicted probability of the body mass index *z*-score by log2 biomarker concentration *z*-scores, holding all other biomarkers at their medians.

**Figure 3:**
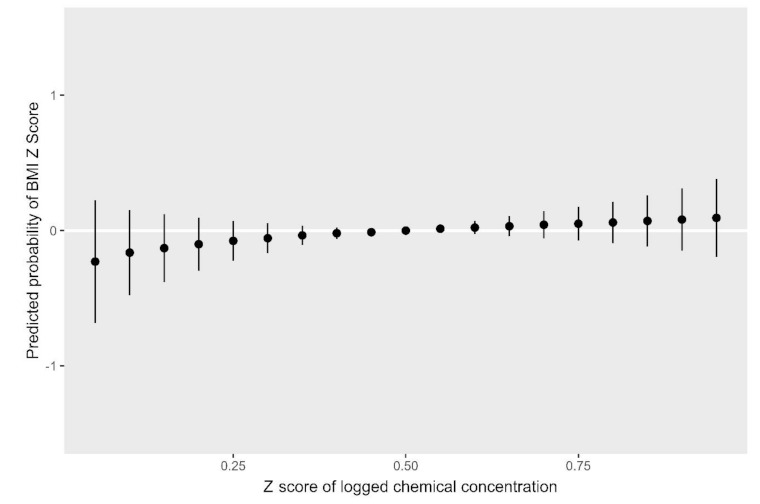
Predicted probability of the body mass index *z*-score by quantiles of total log2 biomarker concentration *z*-scores.

**Figure 4:**
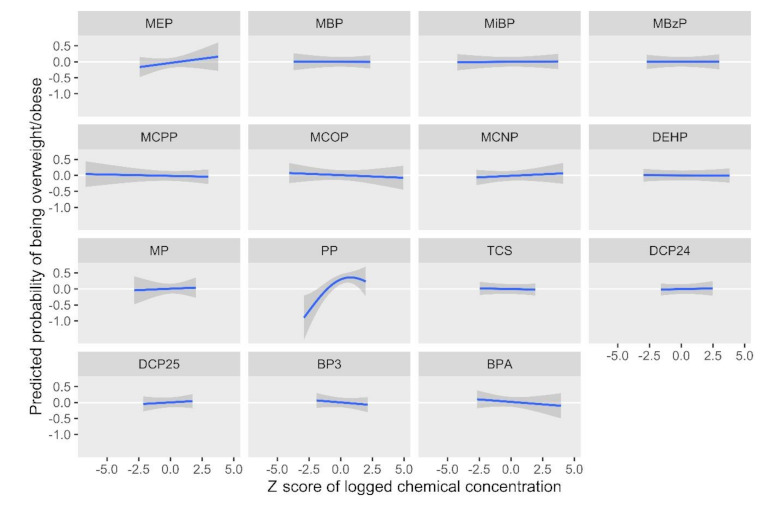
Predicted probability of being overweight/obese by log2 biomarker concentration *z*-scores, holding all other biomarkers at their medians.

**Figure 5:**
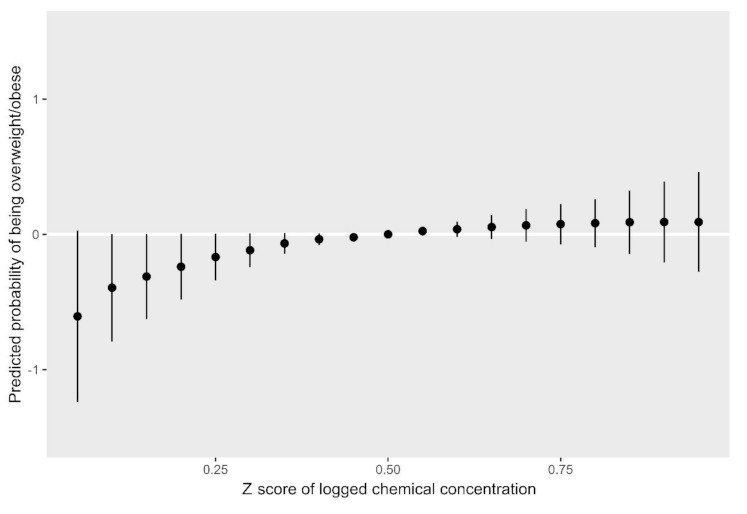
Predicted probability of being overweight/obese by quantiles of total log2 biomarker concentration *z*-scores.

Figure 2 and Figure 3 show results from the BKMR models for the BMI *z*-score. Holding all other biomarkers at their medians, propylparaben, MEP, MBP, and MCNP appeared to be positively associated with the childhood BMI, while MCOP, MCPP, benzophenone-3, and BPA appeared to have an inverse relationship with the outcome (Figure 2). We observed a slight trend of an increasing BMI *z*-score with higher quantiles of all biomarkers; however, credible intervals were very wide (Figure 3). Figure 4 and Figure 5 show BKMR results modeling the overweight/obesity status. Holding all other biomarkers at their medians, propylparaben and MEP were positively associated with the outcome, and we observed a non-linear curve for propylparaben (Figure 4). There was an increase in the predicted probability of being overweight/obese in association with increasing quantiles of all biomarkers (Figure 5); however, credible intervals were again very wide.

When not controlling for fast-food consumption at age five in a sensitivity analysis, results were similar except that the single-biomarker association between propylparaben and the BMI *z*-score was attenuated (β: 0.06, 95% CI: −0.02, 0.10; other data not shown). Results from models not adjusted for any covariates were similar to results from the fully adjusted models (data not shown). Analyses varying the *τ* in BHM models of the BMI *z*-score showed similar results to the main model (data not shown).

## 4. Discussion

We found that prenatal urinary concentrations of MEP, MCNP, and propylparaben showed consistent associations with both the BMI *z*-score and the overweight/obesity status at age five across modeling approaches, all reflecting a relationship with greater childhood adiposity. Associations incorporating multiple pollutants using BHM were somewhat attenuated compared to single-pollutant models, with the exception of MCNP and the overweight/obesity status. The association of MCNP and the overweight/obesity status was only seen with the BHM model, indicating the necessity of accounting for co-pollutant exposure. BKMR models showed similar trends as the single-pollutant and BHM models and indicated propylparaben as having the strongest association with both outcomes; however, credible intervals were very wide. We also observed trends of associations between higher concentrations of all scaled biomarkers in a cumulative mixture and greater adiposity.

Previous studies using single-pollutant models have also mostly found MEP to be associated with childhood adiposity [15] and MCNP to be associated with adult adiposity [49]; however, prior evidence largely suggests a lack of association with propylparaben [17,18,19] and inconsistent associations with other biomarkers analyzed in the current study. There is a wide range of demographic characteristics, geographical locations, and ages in the literature, which may explain some inconsistent results. In our previous single-biomarker analyses of prenatal phthalates and childhood obesity in this cohort, we observed associations of MEP and several measures of adiposity assessed at ages 5, 7, 9, 10.5, and 12 and of MCNP with adiposity outcomes assessed at ages 5, 7, and 9. Associations with MEP, but not MCNP, were robust in BKMR analysis at age 12, which additionally showed negative trends of associations with MCOP and MCPP [33]. Unlike the current study, BKMR in our prior study did not show a positive trend for MCNP, though our previous study examined phthalates only.

The three previous studies that have examined the association of adiposity with similar biomarkers in a mixture context employed different statistical methods, which can affect te interpretation and comparison of results. Agay-Shay et al., the only other study to examine prenatal exposures, analyzed prenatal phthalates and BPA alongside organochlorines, metals, and polybrominated diphenyl ethers in 470 Spanish children aged seven using principle component analysis and found most phthalates to be negatively associated with overweight status, while MEP and BPA were not associated with overweight status [36]. Two studies have examined several of the above biomarkers in the cross-sectional NHANES in relation to obesity. In children and adolescents, a weighted quantile sum (WQS) analysis of phenols, phthalates, and pesticides showed that 2,5-dichlorophenol, BPA, and MEP were positively associated with obesity status but not with the BMI *z*-score [34]. The same study used BKMR and showed a positive association trend between MEP, MiBP, and 2,5-dichlorophenol and measures of adiposity, yet a negative association trend with 2,4-dichlorophenol and methylparaben. In adults, WQS analysis of the same biomarkers showed that MCOP, MEP, and BPA were associated with overall and abdominal obesity, and BKMR showed MCOP to have a positive and nonlinear relationship with obesity [35]. Across these three prior studies and the current study, MEP and BPA have shown the most consistent associations with greater adiposity, though we found a negative association between adiposity and BPA. However, it is difficult to directly compare results across these studies due to the heterogeneity in the biomarkers examined, time points at which children were assessed, and statistical methods employed. Additionally, as seen in our previous study, associations can differ across childhood ages.

While the relationship between propylparaben and both outcomes seen in BKMR plots aligns with its relatively high group and individual PIP values, the relationship seen for MCNP is in contrast with its relatively low PIP values. This may indicate the MCNP relationship should be interpreted with caution, especially considering its wide credible intervals. We observed several inverse associations in BKMR with both the BMI *z*-score and overweight/obesity status, notably for BPA in both outcomes. As seen in the previous paragraph, prior mixture studies have shown this biomarker to be associated with increased adiposity [34,35]. Our discordant results may be due to a lack of power for conducting BKMR with our sample size. BPA was not associated with the BMI *z*-score or overweight/obesity status in single-biomarker or BHM models. In addition, we observed a lack of relationship between several biomarkers and measures of adiposity. This may reflect a true absence of a relationship or could be due to a lack of power, given our sample size, or the difficulty in capturing regular exposure to these biomarkers in two urine samples (see below).

While single-pollutant models are most understandable and interpretable, and are thus an important part of analyzing complex exposure patterns, the relationships among biomarkers and outcomes can be further illuminated by mixture methods that account for potential co-pollutant confounding. Bayesian methods are particularly appealing for mixture analyses, as they can simultaneously model multiple highly correlated exposures [50]. By facilitating a borrowing of information across similar exposures, BHM produces estimates with a lower mean-square error and interval estimate coverage closer to the nominal level, while producing interpretable results in a similar format to those from single-pollutant models [51]. BKMR can also robustly model the relationships of all biomarkers with the BMI or overweight/obesity status simultaneously but can represent that model in several graphical forms. The BKMR outputs allow for a more nuanced understanding of these relationships, but they can be more difficult to interpret. Additionally, because the BKMR credible intervals take into account the uncertainty in multiple steps of the modeling, a greater sample size is generally required to yield significant results.

The few attenuated results seen in our sensitivity analysis excluding fast-food consumption at age five may reflect this covariate’s strong relationship with the outcomes and a loss of precision with its exclusion. Childhood fast food intake may also be a poor proxy for mothers’ prenatal fast-food intake (e.g., children may eat more fast food than their parents did). Though the obesogenic mechanisms of these chemicals are not completely understood, several biological pathways could plausibly mediate the observed relationships [10]. Several phthalates, particularly monoester metabolites, and parabens are known to modulate the activity of peroxisome proliferator-activated receptors (PPAR)-α and PPAR-γ, nuclear receptors that regulate adipocyte differentiation and lipid metabolism [13,52,53]. Prenatal exposure to MEP and other phthalates has been associated with lower thyroid hormone levels in children [54,55], suggesting that disruption of the thyroid’s regulation of energy metabolism could also contribute to adiposity. Some evidence also points to possible epigenetic mechanisms through which prenatal phthalate exposure may influence gene expression and fetal programming [56,57].

Urinary concentrations of chemicals found in consumer products or their metabolites vary widely with the frequency and type of product use [58]; however, people tend to use the same products over time [59]. In addition, most of these chemicals have short half-lives [60], and spot urine samples may not represent exposure throughout pregnancy. Biomarkers of exposure included in the present study had relatively low intraclass correlation coefficients (ICCs), indicating high variability in exposure. ICCs for phthalate metabolites ranged from 0.14 to 0.39, for parabens ranged from 0.41 to 0.46, and for phenols ranged from 0.16 to 0.56. Fortunately, in our study, we were able to collect urine at two time points during pregnancy and then calculated the average, which should provide a better estimate of exposure throughout pregnancy [61].

Strengths of this study include quantification of 15 endocrine-disrupting biomarkers in two urine samples; the use of two mixture methods, which improves the robustness of findings and control for co-pollutant exposure; a prenatal exposure window; and a rich longitudinal cohort with robust anthropometric measures at age five. Limitations include moderately correlated exposures, which may not be entirely accounted for in our methods; high variability in exposure to chemicals with low half-lives; and a lack of power to conduct sex-stratified Bayesian models.

## 5. Conclusions

Using Bayesian mixture methods and single-pollutant models, we found that several biomarkers of chemicals found in personal care and plastic products were associated with increased adiposity at age five, most notably MEP (parent compound DEP), MCNP (parent compound DiDP), and propylparaben. This analysis extends previous research by employing two emerging methods, BHM and BKMR, to examine associations of prenatal exposure with ubiquitous chemical mixtures and childhood adiposity. Results, which were consistent across these methods controlling for co-pollutant confounding, in addition to single-pollutant regressions, suggest that children exposed to DEP, DiDP, or propylparaben prenatally may be at higher risk for obesity.

## Figures and Tables

**Table 1:** Demographic characteristics of the CHAMACOS population (*N* = 309).

Characteristic	*N*	%
**Maternal race/ethnicity**		
Latina	303	98.1
Non-Latina, white	2	0.6
Other	4	1.3
**Maternal age at delivery**		
18–24 years	123	39.8
25–29 years	103	33.3
30–34 years	52	16.9
35+ years	31	10.0
**Maternal education**		
≤6th grade	134	43.4
7th–12th grade	108	34.9
≥High school graduate	67	21.7
**Maternal years of residence in the U.S. at delivery**		
≤5 years	141	45.6
6–10 years	86	27.8
≥11 years	82	26.6
**Household income during pregnancy**		
Below or equal to poverty line	194	62.8
Above poverty line	115	37.2
**Maternal pre-pregnancy BMI**		
Underweight	2	0.6
Normal	104	33.7
Overweight	122	39.5
Obese	81	26.2
**Childhood fast-food consumption at age 5**		
<1 time/week	112	36.2
1–2 times/week	189	61.2
≥3 times/week	8	2.6
**Childhood birth weight**		
< 2500 g	13	4.2
≥2500 g–<4000 g	249	80.6
≥4000 g	47	15.2
**Childhood BMI at age 5**		
Underweight	1	0.3
Normal	142	46.0
Overweight	65	21.0
Obese	101	32.7

BMI: body mass index; CHAMACOS: Center for the Health Assessment of Mothers and Children of Salinas.

**Table 2:** Distribution of uncorrected and specific-gravity-corrected (italics) biomarker concentrations in maternal urine collected at two time points during pregnancy (*N* = 309).

Biomarker	% > LOD		Average of Two Measurements						
	1st Measurement	2nd Measurement	Geo. Mean	10th%	25th%	50th%	75th%	90th%	95th%
**LMW phthalates**									
MEP (ng/mL)	100	100	182.7	34.4	80.8	180.0	413.5	792.0	1252.5
			*236.4*	*46.7*	*105.5*	*225.6*	*501.9*	*953.5*	*1615.9*
MBP (ng/mL)	98	100	21.8	7.8	12.2	20.7	41.6	69.6	96.9
			*27.7*	*10.5*	*16.2*	*26.8*	*46.6*	*89.9*	*117.4*
MiBP (ng/mL)	93	96	2.7	0.8	0.8	2.8	5.2	8.4	17.3
			*3.4*	*1.0*	*1.9*	*3.5*	*6.7*	*11.0*	*16.6*
**HMW phthalates**									
MBzP (ng/mL)	98	99	7.0	1.9	3.8	7.4	13.6	25.8	31.1
			*8.9*	*2.5*	*4.9*	*9.1*	*17.1*	*28.1*	*37.8*
∑DEHP (nmol/mL)	N/A	N/A	0.2	0.1	0.1	0.2	0.3	0.5	0.8
			*0.2*	*0.1*	*0.1*	*0.2*	*0.4*	*0.6*	*0.8*
MEHP (ng/mL)	88	92	3.6	1.0	1.9	3.7	7.1	12.1	19.1
			*4.5*	*1.4*	*2.5*	*4.5*	*7.8*	*15.7*	*19.3*
MEHHP (ng/mL)	100	99	14.9	4.6	8.2	14.9	27.7	46.7	63.4
			*18.8*	*6.6*	*10.8*	*18.7*	*33.4*	*51.8*	*72.6*
MECPP (ng/mL)	100	100	25.5	9.0	15.0	24.3	42.5	67.9	89.9
			*32.3*	*12.2*	*19.9*	*32.1*	*51.0*	*77.2*	*108.8*
MEOHP (ng/mL)	98	99	11.0	3.5	6.4	10.9	20.7	33.5	49.6
			*13.8*	*5.1*	*7.7*	*14.2*	*22.7*	*38.8*	*48.3*
MCPP (ng/mL)	88	94	1.7	0.6	1.0	2.1	3.1	4.5	7.2
			*2.2*	*0.8*	*1.4*	*2.4*	*3.8*	*5.2*	*8.1*
MCOP (ng/mL)	96	96	3.0	1.1	1.7	3.1	5.0	7.2	11.4
			*3.8*	*1.5*	*2.4*	*3.9*	*5.7*	*9.0*	*13.1*
MCNP (ng/mL)	95	97	1.8	0.6	1.2	1.9	3.0	4.3	5.9
			*2.3*	*1.0*	*1.5*	*2.3*	*3.4*	*5.1*	*7.6*
**Parabens**									
MP (ng/mL)	100	100	119.3	20.2	56.9	149.0	328.0	503.0	629.0
			*152.8*	*28.6*	*72.7*	*183.1*	*365.8*	*631.3*	*771.2*
PP (ng/mL)	96	98	30.4	2.2	8.7	36.9	135.9	452.8	523.3
			*38.6*	*2.7*	*10.6*	*46.5*	*164.9*	*429.4*	*631.4*
**Other phenols**									
TCS (ng/mL)	71	75	18.2	1.3	4.2	15.7	104.7	331.0	481.5
			*23.6*	*1.6*	*6.3*	*20.7*	*142.7*	*373.6*	*625.4*
2,4-DCP (ng/mL)	100	100	4.7	1.1	1.7	3.2	11.7	47.4	52.2
			*6.0*	*1.5*	*2.4*	*3.8*	*14.6*	*48.7*	*69.3*
2,5-DCP (ng/mL)	100	100	58.8	5.4	11.3	49.8	406.6	684.0	880.5
			*75.1*	*6.7*	*15.1*	*76.6*	*473.3*	*857.1*	*1101.4*
BP-3 (ng/mL)	100	99	19.4	1.6	3.9	12.8	119.7	502.9	693.0
			*25.4*	*2.5*	*4.8*	*16.7*	*145.0*	*559.4*	*944.4*
BPA (ng/mL)	84	89	1.1	0.4	0.7	1.1	1.9	3.0	4.5
			*1.5*	*0.6*	*0.9*	*1.4*	*2.3*	*3.5*	*5.8*

2,4-DCP: 2,4-dichlorophenol; 2,5-DCP: 2,5-dichlorophenol; BP-3: benzophenol-3; BPA: bisphenol A; DEHP: di(2-ethylhexyl) phthalate; HMW: high molecular weight; LOD: limit of detection; LMW: low molecular weight; MBP: mono-n-butyl phthalate; MBzP: monobenzyl phthalate; MCNP: mono(carboxynonyl) phthalate; MCOP: monocarboxyisooctyl phthalate; MCPP: mono(3-carboxypropyl) phthalate; MECPP: mono-(2-ethyl-5-carboxypentyl) phthalate; MEHP: mono-2-ethylhexyl phthalate; MEHHP: mono-(2-ethyl-5-hydroxyhexyl) phthalate; MEOHP: mono-(2-ethyl-5-oxohexyl) phthalate; MEP: monoethyl phthalate; MiBP: mono-isobutyl phthalate; MP: methylparaben; N/A: not applicable; PP: propylparaben; TCS: triclosan.

**Table 3:** Associations between biomarkers and BMI or overweight/obesity in single-biomarker regression models and Bayesian hierarchical models (*N* = 309).

Biomarker	BMI *z*-Score, *β* (95% CI or CrI)		Overweight/Obesity, OR (95% CI or CrI)	
	Single-Biomarker Models ^1^	Bayesian Hierarchical Models	Single-Biomarker Models ^1^	Bayesian Hierarchical Models
**LMW phthalates**				
MEP	0.10 (0.03, 0.17)	0.08 (0.00, 0.16)	1.23 (1.08, 1.42)	1.20 (1.02, 1.42)
MBP	0.07 (−0.04, 0.17)	0.08 (−0.06, 0.22)	1.02 (0.85, 1.23)	1.00 (0.78, 1.29)
MiBP	0.01 (−0.07, 0.10)	−0.02 (−0.12, 0.09)	1.02 (0.87, 1.20)	1.03 (0.84, 1.26)
**HMW phthalates**				
MBzP	0.02 (−0.08, 0.12)	−0.03 (−0.16, 0.10)	1.05 (0.88, 1.25)	1.02 (0.80, 1.30)
MCPP	−0.01 (−0.12, 0.09)	−0.08 (−0.21, 0.05)	0.95 (0.78, 1.14)	0.85 (0.65, 1.11)
MCOP	0.03 (−0.09, 0.14)	−0.02 (−0.19, 0.15)	0.97 (0.79, 1.19)	0.87 (0.64, 1.18)
MCNP	0.10 (−0.02, 0.23)	0.15 (−0.02, 0.32)	1.18 (0.94, 1.49)	1.37 (1.01, 1.89)
DEHP	0.03 (−0.08, 0.14)	0.02 (−0.11, 0.15)	1.00 (0.82, 1.21)	1.01 (0.78, 1.28)
**Parabens**				
MP	0.08 (0.01, 0.16)	0.02 (−0.09, 0.13)	1.22 (1.06, 1.40)	1.07 (0.88, 1.31)
PP	0.06 (0.02, 0.10)	0.04 (−0.02, 0.10)	1.14 (1.05, 1.24)	1.08 (0.97, 1.22)
**Other phenols**				
Triclosan	−0.02 (−0.06, 0.02)	−0.03 (−0.07, 0.02)	0.97 (0.90, 1.05)	0.94 (0.86, 1.03)
2,4-DCP	0.01 (−0.06, 0.08)	−0.04 (−0.20, 0.12)	1.07 (0.95, 1.22)	1.03 (0.77, 1.37)
2,5-DCP	0.02 (−0.03, 0.06)	0.03 (−0.08, 0.14)	1.05 (0.96, 1.14)	1.03 (0.85, 1.25)
BP-3	−0.01 (−0.05, 0.03)	−0.03 (−0.07, 0.02)	1.00 (0.92, 1.08)	0.97 (0.89, 1.06)
BPA	−0.06 (−0.18, 0.07)	−0.10 (−0.23, 0.04)	0.87 (0.70, 1.09)	0.81 (0.62, 1.04)

2,4-DCP: 2,4-dichlorophenol; 2,5-DCP: 2,5-dichlorophenol; BMI: body mass index; BP-3: benzophenol-3; BPA: bisphenol A; CI: confidence interval; CrI: credible interval; DEHP: di(2-ethylhexyl) phthalate; HMW: high molecular weight; LOD: limit of detection; LMW: low molecular weight; MBP: mono-n-butyl phthalate; MBzP: monobenzyl phthalate; MCNP: mono(carboxynonyl) phthalate; MCOP: monocarboxyisooctyl phthalate; MCPP: mono(3-carboxypropyl) phthalate; MEP: monoethyl phthalate; MiBP: mono-isobutyl phthalate; MP: methylparaben; N/A: not applicable; OR: odds ratio; PP: propylparaben; TCS: triclosan; ^1^ Separate models created for each chemical.

**Table 4:** Individual posterior inclusion probabilities from Bayesian kernel machine regressions (*N* = 309).

Biomarker	BMI *z*-Score (Group PIP = 0.85)	Overweight/Obesity Status (Group PIP = 0.97)
MEP	0.109	0.037
MBP	0.029	0.003
MiBP	0.015	0.003
MBzP	0.020	0.003
MCPP	0.015	0.004
MCOP	0.016	0.006
MCNP	0.026	0.005
DEHP	0.016	0.003
MP	0.168	0.027
PP	0.478	0.878
Triclosan	0.023	0.005
24DCP	0.021	0.008
25DCP	0.022	0.006
BP3	0.020	0.004
BPA	0.021	0.006

## Supplementary Materials

The following are available online at https://www.mdpi.com/1660-4601/18/4/1796/s1: Table S1: Demographic characteristics of the CHAMACOS population, comparing those followed to 5 years vs. lost to follow-up.
