# Supplementary material for: Prenatal Exposure to Mixtures of Phthalates, Parabens, and Other Phenols and Obesity in Five-Year-Olds in the CHAMACOS Cohort
Source: Int J Environ Res Public Health. 2021 Feb 12;18(4):1796. doi: 10.3390/ijerph18041796 (PMC7918439; doi:10.3390/ijerph18041796)
Supplement: Supplementary file 1 [file ijerph-18-01796-s001.pdf]

Table S1. Demographic characteristics of the CHAMACOS population, comparing those followed to 5 years vs. lost to follow-up

	**Followed to age 5 (n=309)**	**Not followed to age 5 (n=292)**	p_{diff}
	**N (%)**		
Maternal race/ethnicity			
Latina	303 (98.1)	275 (94.5)	0.07
Non-Latina, White	2 (0.6)	6 (2.1)	
Other	4 (1.3)	10 (3.4)	
Maternal age at delivery			
18-24	123 (39.8)	124 (53.9)	<0.01
25-29	103 (33.3)	64 (27.8)	
30-34	52 (16.9)	33 (14.4)	
35+	31 (10.0)	9 (3.9)	
Maternal education			
≤ 6th grade	134 (43.4)	127 (43.6)	0.52
7th-12th grade	108 (34.9)	111 (38.1)	
≥ High school graduate	67 (21.7)	53 (18.2)	
Maternal years of residence in the United States at delivery			
≤ 5 years	141 (45.6)	169 (58.1)	<0.01
6-10 years	86 (27.8)	42 (14.4)	
≥ 11 years	82 (26.6)	80 (27.5)	
Household income during pregnancy			
Below or equal to poverty line	194 (62.8)	175 (60.3)	0.54
Above poverty line	115 (37.2)	115 (39.7)	
Maternal pre-pregnancy BMI			
Underweight	2 (0.6)	1 (0.5)	0.01
Normal	104 (33.7)	94 (43.7)	
Overweight	122 (39.5)	81 (37.7)	
Obese	81 (26.2)	39 (18.1)	
Child birth weight			
< 2,500 g	13 (4.2)	15 (5.1)	<0.01
≥ 2,500 g - < 4,000g	249 (80.6)	188 (64.4)	
≥ 4,000 g	47 (15.2)	89 (30.5)	

BMI: Boddy Mass Index; CHAMACOS: Center for the Health Assessment of Mothers and Children of Salinas
